# *R-Loop Tracker*: Web Access-Based Tool for R-Loop Detection and Analysis in Genomic DNA Sequences

**DOI:** 10.3390/ijms222312857

**Published:** 2021-11-27

**Authors:** Václav Brázda, Jan Havlík, Jan Kolomazník, Oldřich Trenz, Jiří Šťastný

**Affiliations:** 1 Institute of Biophysics of the Czech Academy of Sciences, Královopolská 135, 612 65 Brno, Czech Republic; vaclav@ibp.cz; 2 Department of Informatics, Mendel University in Brno, Zemědělská 1, 613 00 Brno, Czech Republic; xhavlik@mendelu.cz (J.H.); jan.kolomaznik@gmail.com (J.K.); oldrich.trenz@gmail.com (O.T.); 3 Faculty of Mechanical Engineering, Brno University of Technology, Technická 2896/2, 616 69 Brno, Czech Republic

**Keywords:** sequence analysis, RNA–DNA hybrid, non-B structure

## Abstract

R-loops are common non-B nucleic acid structures formed by a three-stranded nucleic acid composed of an RNA–DNA hybrid and a displaced single-stranded DNA (ssDNA) loop. Because the aberrant R-loop formation leads to increased mutagenesis, hyper-recombination, rearrangements, and transcription-replication collisions, it is regarded as important in human diseases. Therefore, its prevalence and distribution in genomes are studied intensively. However, in silico tools for R-loop prediction are limited, and therefore, we have developed the *R-loop tracker* tool, which was implemented as a part of the *DNA Analyser* web server. This new tool is focused upon (1) prediction of R-loops in genomic DNA without length and sequence limitations; (2) integration of *R-loop tracker* results with other tools for nucleic acids analyses, including Genome Browser; (3) internal cross-evaluation of in silico results with experimental data, where available; (4) easy export and correlation analyses with other genome features and markers; and (5) enhanced visualization outputs. Our new *R-loop tracker* tool is freely accessible on the web pages of *DNA Analyser* tools, and its implementation on the web-based server allows effective analyses not only for DNA segments but also for full chromosomes and genomes.

## 1. Introduction

In addition to the basic DNA structure first described by Watson and Crick in 1953 [1], new findings show many nucleic acid structures differing from this canonical B-DNA structure. The best-described local non-B nucleic structures are cruciform [2,3], left-handed Z-DNA [4,5], triplexes [6,7], and quadruplexes [8,9]. Contemporary research has demonstrated that these structures are present within the genomes of all organisms and play important roles in many basic biological functions [10,11,12,13]. R-loops are regular three-stranded non-B nucleic acid structures constituted from an RNA–DNA hybrid [14]. The formation of R-loops plays a role in DNA replication, mutations, and homologous recombination, but it also is suggested to be an inducer of trinucleotide repeat expansion associated with human neuromuscular degenerative diseases [14,15]. Furthermore, R-loop resolution defects have been found to be associated with an increasing number of human diseases [16,17]. Negative supercoiling of DNA during transcription facilitates the formation of local nucleic acid structures, including cruciform and R-loop [18,19]. R-loop structures have engendered immense interest due to their involvement in such important biological processes as transcription, mRNA splicing, DNA replication, recombination, and repair [20]. Although there are several tools to study inverted repeats that form cruciform, G-quadruplexes, and other non-B DNA structures in nucleic acids [11,21,22,23,24], in silico tools for the prediction of R-loops are limited [22]. The authors of the QmRLFS-finder tool used a quantitative structural model for an R-loop-forming sequence written in Python-based code [25]. In our *R-loop tracker* implementation, we used a modern Java environment to enhance performance and optimize the algorithm for full genome analyses without length limitations. The *R-loop tracker* tool has been implemented as part of the *DNA Analyser* web server, and this completely new implementation enables server-based prediction of R-loops while integrating its results with other tools for nucleic acids analyses, internal cross-evaluation of results with experimental data, easy export and correlation analyses with other genome features and markers, and enhanced visualization outputs. The *R-loop tracker* tool is integrated into the web pages of *DNA Analyser* tools http://bioinformatics.ibp.cz/ (accessed on 28 October 2021) and freely accessible. 

## 2. Methods and Results

### 2.1. Features

*R-loop tracker* is part of the *DNA Analyser* web server, which provides multiple analyses and sequence operations at a single location. Our implementation utilizes the Python-based QmRLFS finder algorithm [25] and benefits from modern, rapid processing of Java-based code, a web-based interface with rich graphical results presentation, and server data management. The workflow and implementation were completely rewritten for a server-based platform, coded, and constructed so that all imported sequences and analyses are retained in the user database. The server architecture operates in batches and provides an API, which can be used for full genome sequence analyses.

### 2.2. Input and Analysis

Sequences can be imported individually or in larger numbers according to NCBI ID (RefSeq). Another option is to import a sequence via file either in a plain text format or in FASTA format. For quick operations and short sequence tests, there is an option to import a sequence directly from a computer’s clipboard into a web application form. One can also add tags to each sequence to group them according to the project or planned analysis. Upon importing a sequence, it will remain linked to your account so that one can work with the same sequence later at any time. Individual sequence length is limited only by the maximum file upload size, which is currently 2048 MiB. Therefore, one can easily import and analyze whole chromosomes. Such an analysis takes longer to process, of course, and once such an operation is completed, the results will be stored on the server, where they can be found later and displayed from the “Stored results” tab.

Two detection models for the R-loop analysis are available in the “R-loop tracker” tab: The RIZ 3g-cluster is looking for three consecutive guanine clusters with cluster size of at least three guanines (equal to model *m1* in the original implementation), and the RIZ 2G-cluster is looking for two consecutive guanine clusters with cluster size of at least four guanines (equal to model *m2*). The first of these is the default model for an analysis. R-loops are detected on both DNA strands simultaneously. The *R-loop tracker* application creates the complementary strand and analyzes the parallel sequence as well. R-loops on the strand of the imported sequence are marked as +, R-loops from the complementary strand are marked as −.

### 2.3. R-Loop Detection

The detection algorithm is based on a quantitative structural model for an R-loop-forming sequence [25]. This algorithm has been verified by comparison to in vitro experiments [26] and provides satisfactory results for R-loop detection in selected human/mouse genes.

### 2.4. R-Loop Tracker Web Application Output

**Figure 1:**
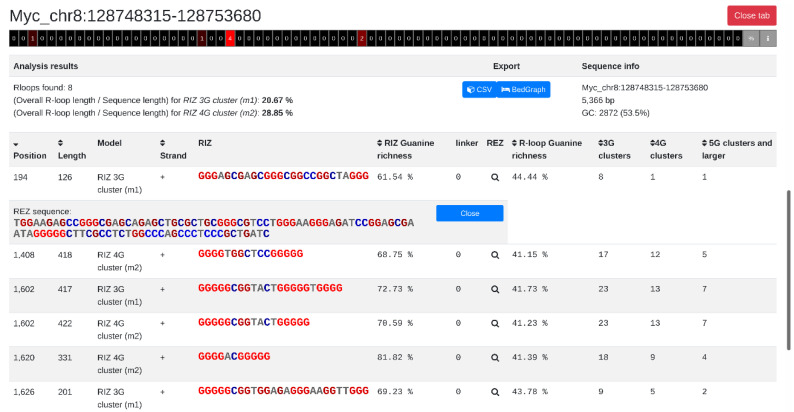
Example of an *R-loop tracker* result, analysis of *Myc* gene location on human chromosome 8.

All results are displayed directly in the browser using AJAX technology. It is possible to run multiple analyses at once using batches and asynchronous events. This ensures that the web server is still able to receive requests while processing analyses in the background. Each analysis is displayed in a separate tab providing detailed information about the detected R-loops (Figure 1). The top part of the tab displays the name of the analyzed sequence and a heat map of R-loop localization in the sequence (showing the number of R-loops found in the location or % of sequence covered by R-loops). Below the heat map, the general sequence information is shown, which includes the number of R-loops found, the R-loop rate throughout the sequence, the sequence length, and the CG content. There are also buttons that provide data export options.

The main windows show *R-loop tracker* results in the form of a table, where every row represents a detected R-loop. The columns provide information about the position of the R-loop in the sequence, R-loop length, model, location of the R-loop according to DNA strand, R-loop initiation zone (RIZ) sequence, guanine density in RIZ sequence, length of the linker, R-loop elongation zone (REZ, which is opened by clicking the magnifier icon), guanine density in REZ sequence, and number of G-clusters based on the G-cluster length. To improve readability and show the important features of R-loop sequences, we formatted the RIZ and REZ sequences based on the numbers of Gs and Cs. The more Gs there are in the cluster, the brighter the intensity of the cluster displayed in red (blue color is used for C clusters). Columns can be sorted by clicking on the arrows in the first row, and therefore, it is very easy to find R-loops with parameters of interest. 

### 2.5. Output Formats

In addition to the graphical representation described above, there is also the possibility to export the analysis data in two different file formats. The first choice is the widely used *CSV* format, which contains the same data as seen in the web browser and is useful for machine processing. The second option is the *bedGraph* format, which can be used for visualization in *Genome Browser* [27]. A *bedGraph* file can be added directly as a custom track into the *Genome Browser* engine. Be aware that this feature requires a specific sequence name for correct integration (see *R-loop tracker* help available on the tool’s web pages). A *bedGraph* file contains a header with information about the analysis and R-loop records consisting of sequence name, start and end position of the R-loop in input sequence, and R-loop score.

The R-loop score reflects the guanine density and cluster occurrences in an R-loop according to the following formula

score = (g_3_ + 2 ∙ g_4_ + 3 ∙ g_5_) ∙ RIZ_G%_ ∙ RLOOP_G%_, (1)

where g_3_, g_4_, and g_5_ represent a guanine cluster count of specific size, and these weighted attributes are multiplied by R-loop initiation zone guanine density (RIZ_G%_) and R-loop guanine density (RLOOP_G%_).

### 2.6. API Usage

*DNA Analyser* provides an application programming interface to integrate one’s scripts or web server with *R-loop tracker*. The API documentation is available at the *DNA Analyser* web site.

## 3. Discussion

R-loops are three-stranded DNA–RNA hybrid molecules that play important roles in a wide range of biological processes, including processes initiating molecular events regulating gene transcription and chromatin modifications [28]. R-loops have also been suggested as hot-spots for double-stranded breaks leading to DNA damage and human diseases [29,30]. These structures are very dynamic, and a subset of R-loops maintained after differentiation has been shown to be associated with repressive chromatin marks on silent pluripotency genes [31]. Meanwhile, several methods for their profiling have been developed in vivo [32,33,34]. Although many tools exist for analyzing other non-B DNA structures in silico, the options for R-loop analyses are limited. Therefore, we integrated a tool for R-loops into our *DNA Analyser* server. This new tool, *R-loop tracker*, is a web application capable of processing multiple analyses simultaneously while having the capability to process large input sequences, including full-length chromosomes. In addition to this advantage, the results are presented in a web browser with enhanced visualization and are sortable, thereby enabling effective information mining from *R-loop tracker* analyses. The web server runs on up-to-date technologies and provides means of integration with other tools and servers. Contemporary DRIP-seq analysis can provide experimental data of R-loop presence in various organisms, in various cell lines, and under diverse conditions [35,36,37]. A comparison of *R-loop tracker* results with DRIP-seq data showed that *R-loop tracker* can help find target sequences in silico. Interestingly, R-loops, and especially the RIZ parts, are also similar to sequences predicted as G-quadruplexes by the G4Hunter algorithm [23]. These results suggest an important role of other non-B DNA structures for effective R-loop formation. On the other hand, the overlap between various R-loop data sets is still poor in some cases. Therefore, future training of the *R-loop tracker* algorithm on an experimental data set can be beneficial. Fortunately, the *R-loop tracker* tool can be easily updated to improve the results and detection algorithms when more validated data sets are available.

## 4. Materials and Methods

### 4.1. Algorithm Validation

To test our implementation of the *R-loop tracker* tool, we created a command line utility to obtain and compare results of different analyses. This utility uses data downloaded from *Genome Browser* which have to be manually transformed into input files. The utility only loads files in a selected folder, proceeds with comparison, and provides a graphical representation. An algorithm output can also be compared with in vitro data sets present in *Genome Browser*. The data set is available in the utility repository: https://github.com/jan-havlik/genome-comparator (accessed on 28 October 2021), and the repository also contains README to manually recreate the process with a different input sequence if needed.

#### Comparison Method

**Figure 2:**
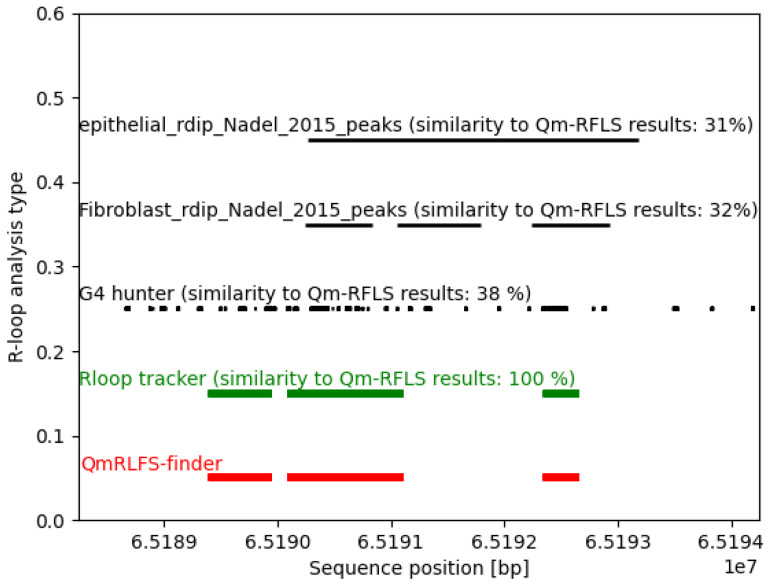
Example of an *R-loop tracker* comparison with QmRLFS finder algorithm on human chromosome 8.

Every interval defined with an R-loop start position and an R-loop end position was expressed as a discrete interval (set) of numbers. These numbers were merged into one set of indices representing every nucleic base occurring in the detected R-loops. We then compared those positions across all methods and we present them here in graphic form. This method of comparison does not take into account the R-loop direction, which may cause irregularities when comparing in vitro results with in silico results. Comparison of analysis results was made using the first 6000 bp in the sequence of the human gene NEAT1 (Figure 2). Within this 6000-bp sequence, the R-loop tracker detected 20 R-loops in three main regions. Comparison with available data sets from the R-loop DB [25] and RDIP (RNA:DNA immunoprecipitation) [38] data sets downloaded from Genome Browser showed that the results of the R-loop tracker were identical to those of the QmRLFS mapper, thus confirming the correctness of the algorithm. The overlay with the Fibroblast RDIP-seq data set was 32% and was located in similar regions of this sequence. Interestingly, the comparison with the G4Hunter results (G4Hunter analysis parameters—windows 25, G4Hunter score > 1) showed a 38% overlap of R-loops with G-prone sequences.

### 4.2. Validation

To validate our implementation, we decided to compare the output of an algorithmic detection with the experimental sequencing method DRIPc [35]. The choice was made due to the availability of the source data in *Genome Browser*, which can be accessed via the API. To validate the effectiveness of the tool, we defined the following metrics for result comparison.

- **TP** (true positive)—at least one R-loop was detected both by DRIPc sequencing and the R-loop tracker algorithm.
- **TN** (true negative)—not a single R-loop was detected with the experimental method nor with the R-loop tracker algorithm.
- **FP** (false positive)—DRIPc sequencing did not detect any R-loop in a given area, but the R-loop tracker found at least one R-loop in a given area.
- **FN** (false negative)—at least one R-loop was detected by DRIPc sequencing but none was found by the R-loop tracker in a given area.

The choice of gene sequences for the comparison was based on previously published fourteen verified genes containing R-loops [25]. The source data and the comparison script are available on GitHub. We measured the following metrics to validate the data for the R-loop tracker:

1. Accuracy
2. Sensitivity
3. Specificity
4. Precision
5. Matthews Correlation Coefficient

We have compared the results for both positive and negative strands, and the results differed by the false positive/false negative ratio (Table 1).

R-loop tracker showed 71.4% in accuracy, 88.9% in sensitivity, and 75% positive Matthews Correlation Coefficient. The validation dataset is available in Supplementary Material online at https://github.com/jan-havlik/validation_dataset (accessed on 28 October 2021).

### 4.3. R-Loop Tracker Effectivity

**Figure 3:**
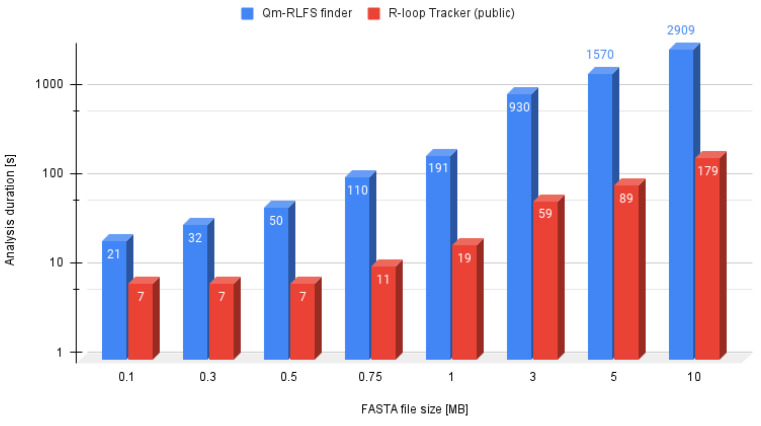
R-loop tracker web server effectivity compared to that of the QmRLFS finder tool.

The effectivity of the R-loop tracker tool was measured by comparing the processing speed of each analysis (Figure 3). The input sequence was split into smaller batches of the following size:

- 100 kB
- 300 kB
- 500 kB
- 750 kB
- 1 MB
- 3 MB
- 5 MB
- 10 MB

Because the QmRLFS finder website only allows files of maximum size of 300 kB, we were using the available command line tool written in Python 2.7. 

As we can see in the figure, R-loop tracker was faster compared to QmRLFS finder. This was also caused by greater computational resources of the standalone server which is publicly accessible to everyone. The time difference was taken using two different approaches:

- Linux *time* utility measuring the script run time
- Time difference calculation from web server logfile

The data set for comparison is available at https://github.com/jan-havlik/comparison (accessed on 28 October 2021).

## Figures and Tables

**Table 1:** Standard performance measure for both strands.

	Positive Strand	Negative Strand
*Accuracy [%]*	78.57	64.29
*Sensitivity [%]*	25	40
*Specificity [%]*	100	77.78
*Precision [%]*	76.92	70
*Matthews Correlation Coefficient*	0.44	0.19

## Data Availability

The tool is available at: https://bioinformatics.ibp.cz/ (accessed on 28 October 2021), the data are available at: https://github.com/jan-havlik/comparison (accessed on 28 October 2021).

## Supplementary Materials

The validation dataset is available online at https://github.com/jan-havlik/validation_dataset.
